# Exploring the protein universe with distant similarity detection methods

**DOI:** 10.1002/pro.70397

**Published:** 2025-12-23

**Authors:** Harutyun Sahakyan, Pascal Mutz, Victor Tobiasson, Eugene V. Koonin

**Affiliations:** ^1^ Computational Biology Branch, Division of Intramural Research National Library of Medicine, National Institutes of Health Bethesda Maryland USA

**Keywords:** distant homology detection, protein universe, sequence alignment, structural search, structure alignment

## Abstract

During the last few years, the body of data on proteins is expanding almost exponentially with the development of advanced methods for gene sequencing, protein structure determination, particularly by cryoelectron microscopy, and structure prediction using artificial intelligence‐based approaches. These developments create the potential for a comprehensive exploration of the protein universe, the entirety of the proteins existing in the biosphere. Elucidation of the relationships among proteins including the most distant ones, where only the core fold is shared, is crucial for understanding protein functions, folding mechanisms, and evolution, as well as the evolution of cellular life forms and viruses. In this brief review, we discuss methods that shaped the field of protein bioinformatics, first, through comparative sequence analysis, and the recent developments in protein structure prediction that transformed the state of the art in the comparative analysis of distantly related proteins. The combination of the rapidly growing databases of genome and metagenome sequences with sensitive methods for sequence comparison and the new generation of structure analysis tools can make charting the protein universe at the structural level a realistic goal.

## 1 INTRODUCTION

Our understanding of proteins and the protein universe, that is, the entirety of proteins that exist in the biosphere (Kolodny et al. 2013; Koonin et al. 2002; Ladunga 1992; Levitt 2009), is advancing quickly with the progress of the theoretical and empirical knowledge driven by multiple methodological breakthroughs over the last decades. Apart from direct experiments, enormous amount of information on protein structure, functions and evolution can be inferred by computational approaches including comparison of protein sequences and experimentally solved or predicted structures. Current protein databases, such as Uniprot or the NCBI protein database, include hundreds of millions of unique sequences, with accompanying information on structure, functions, interactions, evolutionary relationships, and other types of data (Sayers et al. 2025; The UniProt Consortium 2025). The overarching principle of comparative protein analysis is the biological relevance of evolutionary conservation whereby sequence and structure features that are conserved through long evolutionary spans are maintained by purifying selection due to their importance for functions that are shared by proteins in a family. Conversely, fast‐changing portions of proteins that can be subject to positive selection are informative for understanding adaptations, for example, those driven by host–parasite arms races.

The first phase of the protein universe exploration started with the development of efficient methods for gene sequencing followed by whole genome and next generation sequencing, which made protein sequences massively available, paralleled by efficient and robust computational methods for sequence analysis (Altschul et al. 1990; Altschul et al. 1994; Altschul et al. 1997; Altschul and Koonin 1998; Lipman and Pearson 1985; Pearson and Lipman 1988; Wilbur and Lipman 1983). Functions of proteins are directly linked to their structures, rather than to linear sequence, and the second major foray into the protein universe came with the developments of the AI‐based protein structure prediction tools pioneered by AlphaFold2, which predict single‐chain protein structures with near experimental accuracy for the majority of natural protein sequences (Abramson et al. 2024; Baek et al. 2021; Jumper et al. 2021; Krishna et al. 2024; Lupas et al. 2021). The genome‐scale protein structure predictions resulted in the AlphaFold Protein Structure Database (AFDB) (Varadi et al. 2024), and further analysis of these structural models brought to light numerous large (super)families of conserved proteins, often with uncharacterized functions (Lau et al. 2024).

**FIGURE 1:**
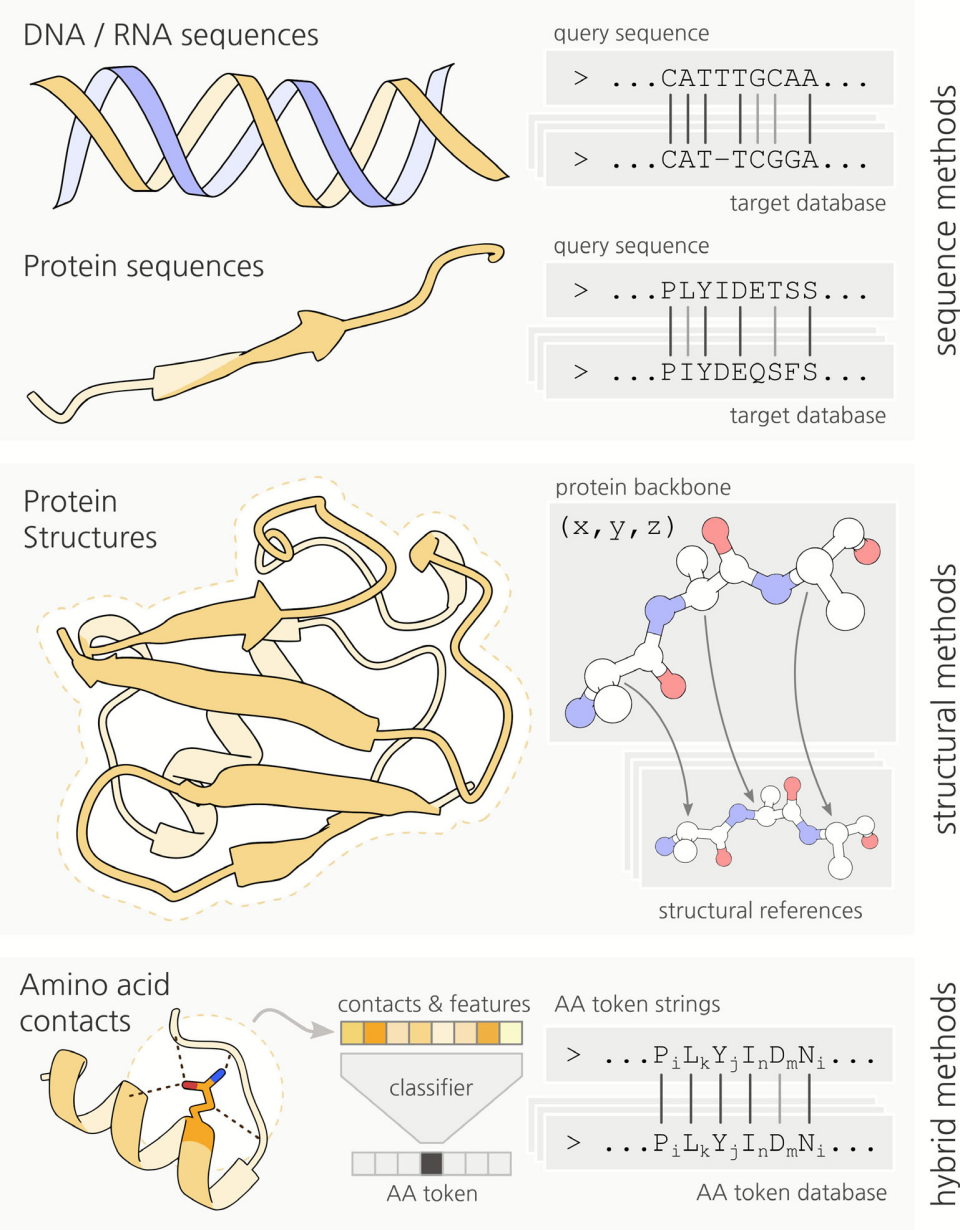
Different approaches for protein comparison. Sequence comparison methods involve searching for an alignment where homologous residues occupy the same positions. Structure search is based on the superimposition of 3D coordinates (or contact maps) to find structurally similar regions. The hybrid methods represent protein structure as a sequence encoding structural information.

Detection of homologous proteins with low sequence similarity is an important and challenging problem in biology that is crucial to protein structure prediction, understanding the relationship between protein sequence, structure, and function, and ultimately, unveiling the evolutionary history of life (Figure 1). Efficient tools for protein sequence comparison, such as FASTA (Lipman and Pearson 1985) and BLAST (Altschul et al. 1990), propelled the development of bioinformatics and dramatically advanced the boundaries of our knowledge about protein structure, function, and evolution. The next generation of tools based on protein family profiles attained substantially enhanced sensitivity in the detection of distant protein homologs by incorporating information about residue conservation from multiple sequence alignments. Position‐specific score matrices (PSSM) implemented, in particular, in Position‐Specific Iterating BLAST (PSI‐BLAST) (Altschul et al. 1997) contain information on the probabilities of amino acids at different positions of multiple sequence alignments (MSA), allowing far more sensitive identification of conserved patterns in the target sequences compared to the original version of BLAST that used single sequences and standard matrices for amino acid comparison. Hidden Markov models (HMM) derived from MSAs additionally contain information about deletions and insertions. HMM against HMM database search, combined with information about secondary structures, implemented, in particular, in the HH‐suite software package of bioinformatics tools (Soding 2005; Zimmermann et al. 2018), is one of the most sensitive approaches for detecting distant similarities among homologous proteins although HMM search against sequence database is also highly effective (Steinegger et al. 2019).

Throughout the evolution of life, protein tertiary structure is typically far more strongly conserved than the amino acid sequence (Illergard et al. 2009) such that structural similarity is often readily detectable between proteins that cannot be identified as homologs with any available sequence comparison methods, including HMM to HMM comparison. For decades, however, the utility of structural comparisons has been severely limited by the paucity of experimentally solved structures and the low accuracy of protein structure modeling. Both problems were solved recently, opening up unprecedented possibilities for exploring the protein universe.

The expanse of the protein universe remains dark, and for understanding its global architecture and limits, accurate and sensitive protein comparison on an increasing scale is indispensable. Furthermore, millions of new sequences are deposited in databases every year, and these include numerous uncharacterized proteins without an obvious relationship to known ones. Many protein families remain completely uncharacterized. Of the vast number of diverse proteins for which sequences are now available, only a small fraction can be studied experimentally. Therefore, protein comparison approaches, even if limited in resolution when low‐sequence identity proteins are compared, remain central to the understanding of protein functions and evolutionary relationships. Here, we present a brief historical overview of protein sequence analysis and then discuss recent developments in protein comparison methods and some popular tools with their advantages and limitations.

## 2 PROTEIN SEQUENCE COMPARISON

The idea that protein sequences could serve as documents of evolutionary history apparently was proposed for the first time in the seminal 1958 article of Crick (Crick 1958). Actual protein sequence comparison started off in the early 1960s, in parallel with sequencing of multiple proteins, at the time, by direct chemical methods. Zuckerkandl and Pauling (Zuckerkandl and Pauling 1965), and independently, Margoliash (Margoliash 1963), and Dayhoff and colleagues (Dayhoff et al. 1983; Eck and Dayhoff 1966) showed for the first time that conservation of protein sequences spanned many millions of years of evolution, and therefore, the decay of sequence similarity with species divergence time can inform evolutionary reconstruction. At the same time, Dayhoff and colleagues made the crucial step of creating the first, manually compiled protein sequence databases and later developed matrices for amino acid comparison (Dayhoff et al. 1983). Obviously, however, with the meager amount of sequences available, these studies did not go much farther than proof of principle.

In the 1970s, as sequence databases started growing faster, through progressing nucleic acid sequencing, the first efficient computational methods for sequence comparison and detection of significantly similar sequence regions were developed; in particular, dynamic programming algorithms of Needleman‐Wunsch (Needleman and Wunsch 1970) and Smith‐Waterman (Smith and Waterman 1981) for the construction of optimal global and local pairwise sequence alignments, respectively. Computational methods for aligning sequences based on these algorithms make use of scoring systems employing amino acid substitution matrices, such as the PAM or BLOSUM series, and a gap‐penalty score (Henikoff and Henikoff 1992; Trivedi and Nagarajaram 2020).

The fast accelerating growth of sequence databases that began in the late 1970s after the invention of Sanger sequencing (Hagen 2011; Sanger et al. 1977; Sanger et al. 1982) made it imperative to substantially increase the speed of sequence similarity searches. Tools such as FASTA (Lipman and Pearson 1985) and BLAST (Altschul et al. 1990) traded some accuracy for a dramatic increase in the search speed (compared to full dynamic programming algorithms) by using k‐mers and heuristic algorithms to find the near‐optimal local pairwise alignment. A crucial achievement of BLAST was the introduction of rigorous statistics based on the extreme value distribution that allowed estimation of the statistical significance of the obtained alignments, the Expect (E) value. However, a major limitation of the original version of BLAST was that only local pairwise alignments without gaps could be produced. Subsequent development extended the algorithm and the statistics to gapped alignments, substantially increasing the resolution power (Altschul et al. 1997). These methods were capable of confidently identifying homologous proteins with pairwise sequence identity above 20–40% depending on the sequence length and amino acid composition (Figure 1) (Rost 1999).

**FIGURE 2:**
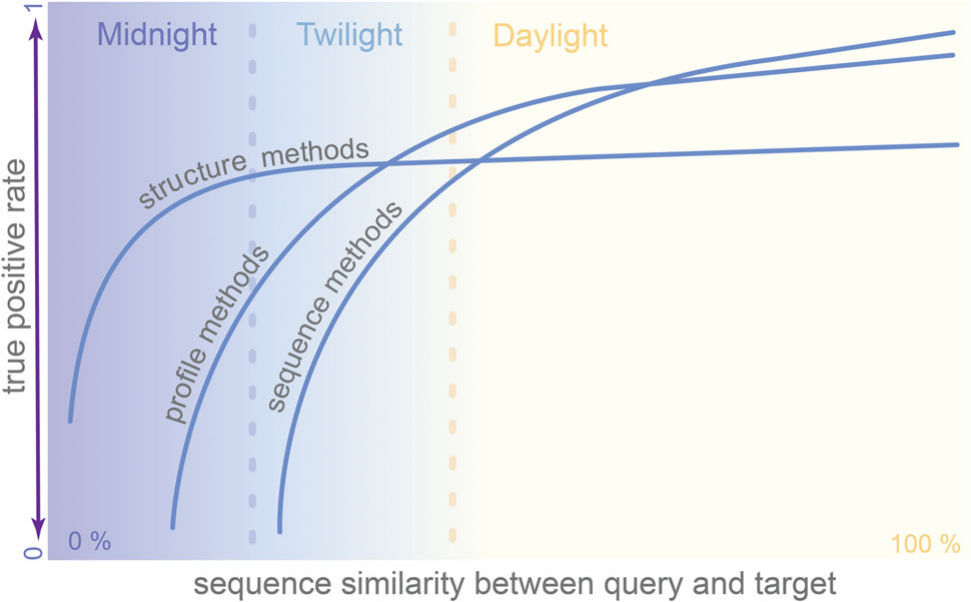
A conceptual plot illustrating how search sensitivity changes depending on the method used and sequence identity. The y‐axis reflects the sensitivity in the same fold or superfamily detection task. Usually, homologous proteins sharing sequence identities above 40% can be confidently identified with sequence alignment tools such as BLAST. In this safe zone, sequence alignment performs much better than structure comparison, especially for small proteins, where structural similarity could result from convergent evolution of non‐homologous proteins into the same fold. Sequence identity of 20–35% is the “Twilight zone” because the accuracy of sequence‐based homology detection methods drops drastically, and the alignments should be inspected carefully. Profile comparison models and structure comparison have higher accuracy in the detection of homologs with twilight identity. Homologs below the Twilight zone can still be detected with HMM‐HMM methods, although with lower accuracy (Soding 2005). Structure comparison methods, however, are more efficient for the comparison of proteins sharing sequence similarity below 20% (Holm 2020; Holm 2022; Holm et al. 2023; Illergard et al. 2009).

In the “twilight zone” (20–35% sequence identity; Figure 2) (Rost 1999), pairwise sequence alignments become unreliable but evolutionarily conserved patterns from multiple sequence alignments can be used to efficiently identify families of distantly related proteins and their functionally important motifs such as catalytic sites of enzymes (Altschul et al. 1997; Bucher et al. 1996; Eddy 1998; Gribskov et al. 1987; Tatusov et al. 1994; Yi and Lander 1994). Within a single amino acid sequence, there is only limited information on the physicochemical properties (e.g., charge, hydrophobicity) and no information on the conservation of each amino acid at a given position across a family of homologous proteins. Hence, in sequence‐to‐sequence comparisons, every pair of matching amino acids is weighted uniformly for all proteins based on universal amino acid comparison matrices such as PAM and BLOSUM. In contrast, multiple sequence alignment allows the identification of conserved residues in a group of homologous sequences and weighs each position individually based on the conservation pattern when running profile‐sequence comparisons. In particular, PSI‐BLAST constructs a MSA on the fly and updates the PSSM used for the database search at each iteration. As a result, the initial alignment can change through the search iterations, typically, resulting in improved accuracy (Chatzou et al. 2016). A conceptually similar but even more powerful approach involves conversion of MSAs to Hidden Markov models (HMMs) that are then used to search sequence databases with high sensitivity as implemented in HMMER (Eddy 1998; Eddy 2011) and the particularly sensitive and fast HHsearch (Soding 2005; Steinegger et al. 2019).

Several powerful, continuously updated algorithms and stand‐alone software also exist for constructing MSAs, including FAMSA (Deorowicz et al. 2016), MAFFT (Katoh et al. 2002), MUSCLE (Edgar 2004; Edgar 2022), HAlign (Zhou et al. 2024), and others, which assign a score for each position based on the evolutionary conservation of the corresponding amino acids, often trading speed for sensitivity or vice versa. The latest versions of some of these methods, in particular, FAMSA and HAlign are capable of aligning many thousands or even millions of protein sequences with acceptable accuracy in a matter of minutes. MSAs generated by these tools can be converted into PSSMs or HMMs and used as queries for the corresponding methods for sequence database search.

As in the case of sequence comparison, comparison of protein family profiles is based on MSAs constructed with different methods and relying on specific scoring systems as well as incorporating secondary structure states as an additional source of information (Soding 2005; von Ohsen et al. 2003). Like sequence alignment methods, algorithms for profile‐profile comparison have substantially advanced since their introduction in the early 2000s, accommodating the needs for increased speed and/or sensitivity. For example, the speed of HMMER3 searches increased about 100 to 1000‐fold compared to HMMER2 by implementing the “multiple segment Viterbi” algorithm (Eddy 2011). Furthermore, a vectorized implementation for the Viterbi algorithm and other speed‐ups have been developed to further increase the efficiency of HHM profile alignment and comparison (Steinegger et al. 2019).

### 2.1 Clustering proteins by sequence similarity

Keeping up with the current load of sequence data makes fast and efficient methods for clustering many millions of proteins by sequence similarity indispensable. Such clustering provides for grouping homologous proteins with flexible similarity thresholds and selecting representatives to construct non‐redundant datasets. Although, in principle, such tasks could be conducted using the tools for protein sequence analysis mentioned above, several dedicated methods have been developed to (pre‐)cluster sequence data in a reasonable time using only modest computational resources (Li et al. 2012; Steinegger and Soding 2018). Among the most popular and thoroughly explored tools are mmseqs2 (Hauser et al. 2016; Steinegger and Soding 2018), CD‐HIT (Fu et al. 2012), UCLUST (Edgar 2010), and DIAMOND (Buchfink et al. 2021). These methods prefilter the dataset by comparing *k*‐mers (substrings of the original sequences of length *k*) to rapidly identify similar sequences, which are then aligned and assigned to a cluster. While the prefilter heuristics implemented in CD‐HIT and UCLUST scale nearly quadratically with the number of compared sequences, the Linclust algorithm implemented in mmseqs2 scales linearly with the number of compared sequences (Steinegger and Soding 2018) and therefore allows clustering of billions of sequences. FLSHclust is another algorithm that scales linearly and, although slightly slower than Linclust, allows clustering of proteins with much lower sequence similarity, as well as distributed computation on clusters without shared memory or disk (Altae‐Tran et al. 2023). DIAMOND is an aligner optimized for very large datasets that runs pseudo‐linearly as well and achieves high performance through the adaptation of its algorithms to up‐to‐date computer architectures and high‐performance clusters/cloud computing (Buchfink et al. 2021; Buchfink et al. 2023).

Even the most sensitive profile‐based methods can fail to detect extremely distant relationships between proteins. Machine learning approaches, some including protein structure information extracted directly from the sequences without intermediate structure prediction, are currently being explored to push the boundaries further (Hamamsy et al. 2024; Routray et al. 2022). Nevertheless, direct protein structure comparison is a more effective approach for identifying distant protein relationships. Below, we discuss several concepts and methods for detecting homology via structural similarity as well as possible future directions.

## 3 PROTEIN STRUCTURE COMPARISON TO DETECT DISTANT HOMOLOGY

Functions of proteins are directly connected with their structures, and throughout evolution, while protein sequences change dramatically, protein folds remain far more conserved (Chothia and Gerstein 1997; Illergard et al. 2009). For instance, in viral proteins, in particular, proteins involved in the virus‐host arms race, and reciprocally, in host immune proteins, amino acid substitutions occur so rapidly that sequence similarity decays beyond recognition whereas the protein fold sustains only minor modifications (Holm 2022; Holm et al. 2023; Krupovic et al. 2022; Mutz et al. 2023; Rajapaksa et al. 2023). In such cases, even with state‐of‐the‐art sequence profile comparison methods, homology often remains undetectable. In contrast, the similarity between distant homologs with extremely diverged sequences can often be detected by structure comparison.

Historically, structure‐based distant homology search was limited by the small number of available experimentally solved structures. The available sequences outnumbered the structures by several orders of magnitude, thanks to the advanced sequencing methods that enable the extraction of protein sequences from numerous genomes and metagenomes. The lack of accurate and fast computational methods for protein structure prediction for many years has been the principal limitation for using structures to infer and explore distant homology. AlphaFold2 (AF2) and RoseTTAFold, both becoming available in 2021, made revolutionary improvements in protein structure prediction using deep learning and taking advantage of evolutionary information mined from protein databases (Abramson et al. 2024; Agard et al. 2022; Baek et al. 2021; Jumper et al. 2021). In particular, AF2 allowed structure prediction for single protein chains with accuracy comparable to experimental methods for the majority of the available protein sequences, and was used to predict the structures of more than 200 million proteins from UniProt, resulting in the AlphaFold Structural Database (AFDB) (Varadi et al. 2024) (https://www.alphafold.ebi.ac.uk/). The more recently released AF3 further increased the accuracy and speed of structure prediction (Abramson et al. 2024). A limitation of AF (here and further we will use AF referring to both versions, AF2 and AF3), however, is that it explicitly relies on the information extracted from a multiple alignment of the query sequence with its homologs that AF detects at a preliminary search step. AF predictions for proteins with no or few detectable homologs typically have low accuracy.

The next important development came from the incorporation of protein language models in the structure prediction workflow. First implemented in ESMfold (Lin et al. 2023), a protein language model was used to provide evolutionary information about conserved and coevolving residues from a single protein sequence instead of an explicit search for this information by searching sequence databases for homologs, which is the rate‐limiting step in AF. Thus, ESMfold and similar approaches based on protein language models allow protein structure predictions for proteins without homologs detectable by sequence comparison, substantially increasing the scope of structure analysis. Furthermore, ESMfold is dramatically faster than AF, and as such, has been used to predict structures of 772 million proteins from the MGnify metagenomic database that are available in the ESM Metagenomic Atlas (https://esmatlas.com/). There is a trade‐off between the general applicability and high speed of ESMfold and its performance in structure prediction compared to AF. A recent study has shown that about 18% of the protein models with low‐confidence ESMfold predictions (pLDDT <60) could be “rescued” by AF2 which produced high‐confidence models for the respective proteins (Yeo et al. 2025). Thus, AF and ESMfold are, to some extent, complementary approaches, and in effect, between these two methods, structural models sufficiently accurate for informative comparative analysis are now available or can be readily obtained for nearly all globular proteins with known sequences.

### 3.1 Protein structure prediction, comparison, and alignment

With the problem of protein structure prediction effectively solved, comparison of structures to identify significant similarities that can be indicative of homology and help reveal functionally important sites that elude sequence searches becomes the key step in the study of the protein universe. A protein structure can be represented in several ways, and based on the type of representation, the majority of approaches for protein structure comparison and alignment belong to one of the following three classes: (i) superimposition of the Cartesian coordinates, (ii) alignment of distance matrices, or (iii) alignment of sequences of structural elements or other embeddings representing the protein structure (Table 1). All these methods usually operate with C_α_ atoms, which are sufficient to represent the protein fold and reduce the amount of data, accelerating the calculations.

**TABLE 1:** Methods for protein structure comparison and alignment.

Method	Protein representation	Web server	Alignment of protein multimers	Multiple structure alignment	Alignment of complexes (including RNA/DNA)	GPU support
Dali (Holm and Sander 1993)	Distance matrix	ekhidna2.biocenter.helsinki.fi/dali	No	No	No	No
TM‐align (Zhang and Skolnick 2005)	C_α_ coordinates	zhanggroup.org/TM‐align	No	No	No	No
GTalign (Margelevicius 2024)	C_α_ coordinates	No	No	No	No	Yes
US‐align (Zhang et al. 2022)	C_α_ coordinates	zhanggroup.org/US‐align	Yes	Yes	Yes	No
SPfast (Litfin et al. 2025)	C_α_ coordinates	No	No	No	No	No
Foldseek (van Kempen et al. 2024)	Structural alphabet	https://search.foldseek.com/search	Yes	Yes	No	Yes
Reseek (Edgar 2024)	Structural alphabet	https://reseek.online/	No	Yes	No	No
pLM‐BLAST (Kaminski et al. 2023)	Sequence embedding	https://toolkit.tuebingen.mpg.de/tools/plmblast	No	No	No	Yes*
TM‐vec (Hamamsy et al. 2024)	Sequence embedding	No	No	No	No	Yes*
PLMSearch (Liu et al. 2024)	Sequence embedding	https://dmiip.sjtu.edu.cn/PLMSearch	No	No	No	Yes*
Progres (Greener and Jamali 2025)	Graph embedding	https://progres.mrc‐lmb.cam.ac.uk/	No	No	No	Yes

* indicate methods that use GPU for database preparation.

In a simple case, protein structures represented as a set of 3D Cartesian coordinates can be aligned by rotation and translation of these coordinates to minimize the root‐mean‐squared deviation (RMSD) between the aligned atoms and find the best fit (Kabsch 1978). This operation is usually performed using the Kabsch‐Umeyama algorithm, which allows calculation of the rotation matrix for minimizing the RMSD between two paired sets of points in 3D space (Kabsch 1976; Umeyama 1991). This procedure works well for proteins with relatively high structural similarity, especially if information from the sequence alignment can be used to find equivalent pairs of atoms and create the initial structural superposition for further optimization. A similar approach is implemented in many molecular graphics visualization tools, for example, in the matchmaker function in ChimeraX (Meng et al. 2006) or the align function of PyMol (DeLano 2002), which allows superposition of the visualized structures. However, if sequences share low similarity and the sequence alignment does not provide necessary information for building an initial alignment, the problem becomes more challenging, and more time is needed to find the correct superimposition. Moreover, the RMSD between atoms, often used as a metric showing the quality of the alignment, might be deceptive when measuring the similarity between two proteins of different size that do not share high structural similarity. The RMSD is sensitive to the length of the compared proteins; minor differences in the positions of aligned amino acid residues can result in a high RMSD even if the global topology is the same, so that large proteins can have high RMSD values despite close structural similarity (Betancourt and Skolnick 2001; Zhang and Skolnick 2005). Thus, for more complicated problems such as searching a database of structures or clustering proteins by structure similarity, this approach does not perform well, and to address these issues, multiple other alignment and scoring strategies have been developed.

DALI, originally developed in 1993 (Holm and Sander 1993; Holm and Sander 1995) and updated through multiple iterations since then, remains one of the most powerful methods for protein structure comparison and was used to discover and analyze evolutionary connections for numerous groups of homologous proteins that were beyond the reach of sequence comparison (Holm 2022; Holm et al. 2023; Holm and Sander 1996). DALI employs an approach based on the alignment of distance matrices instead of explicitly aligning protein coordinates by rotation and translation. The 3D structure of a protein can be represented as a 2D distance matrix containing information about pairwise distances between atoms, which is sufficient to reconstruct the original protein structure (except for its chirality). In the DALI algorithm, the matrices of the target and the query are divided into smaller submatrices to find overlapping fragments, and then, other interconnected fragments are identified using a heuristic search to extend the initial alignment. The aligned fragments are assembled into a global alignment after removing the indels and reordering, revealing similarity between the superimposed structures regardless of gaps or reshuffling of the sequence segments.

The DALI algorithm searches for the optimal superposition of the target and the query structures with the maximum number of overlapping residues with low distances between them, ensuring the best fit. The similarity of aligned structures is evaluated by the DALI score, which is a weighted sum of similarities of intramolecular distances (Holm and Sander 1993). The final DALI Z‐score is normalized by the geometric mean of the lengths of the query and the target although the Z‐score does not have a fixed range and thus can be higher for larger proteins than for shorter ones at the same level of structural similarity. In the absence of rigorous statistics for structural similarity, the significance of the DALI scores is assessed from the results of benchmarking on known protein families. According to the author's recommendation, structures with a Z‐score above 20 are considered homologous; those with Z‐score from 8 to 19 indicate a high probability of homology; those in the Z‐score range from 2 to 8 should be considered candidates for homology that need to be further analyzed, whereas Z‐scores below 2 are considered non‐significant (Holm 2020). Although DALI provides aligned coordinates, its scoring function is not designed for rigid body superimposition. DALI implements an elastic similarity score using relative distance deviations to avoid the cumulative effect of geometrical distortions. Elastic scoring considers the information about secondary structures extracted by DSSP and allows more freedom for helix–helix or helix‐strand interactions than for interactions between adjacent strands forming a beta‐sheet. Thus, some large deviations in absolute space, such as helix displacement, beta‐sheet twisting, or hinge motions, can be compensated for if a good fit in relative space is possible, that is, separate parts of proteins are readily alignable.

TM‐align is a sequence‐independent algorithm for protein structure alignment that is actively used to solve a wide range of problems in protein structure analysis (Zhang and Skolnick 2005). In contrast to DALI, TM‐align uses explicit rotation and translation of 3D coordinates of C_α_ atoms to align structures. C_α_ atoms are also used to define the secondary structures by a built‐in algorithm. Secondary structures represented as a sequence are aligned using dynamic programming to create an initial matching of residues, which is later optimized with a heuristic iterative algorithm. Template modeling (TM)‐score is an essential component of this approach and is necessary for the evaluation of the alignment quality. TM‐score is a variation of the Levitt‐Gerstein weight factor that prioritizes pairs of residues located at closer distances in 3D space, with a higher weight than those at larger distances, in contrast to RMSD, where all pairs are weighted uniformly (Levitt and Gerstein 1998). The final score is normalized by the length of the shorter protein; therefore, regardless of the sizes of the query and target, the TM‐score is always in the range from 0 to 1, where a score higher than 0.5 often indicates that proteins share the same fold and are expected to be classified in the same fold category in the CATH and/or SCOP databases (Xu and Zhang 2010).

The TM‐align approach was extended to TM‐score_RNA_ which aligns RNA structures using C3′ atoms instead of C_α_ (Gong et al. 2019). Then, the universal scoring system allowed the development of US‐align (universal structure alignment) for the superimposition of complexes consisting of proteins, RNA, and DNA, including homooligomeric complexes with high‐order symmetry and mixed heteromeric complexes. In addition to the monomeric and oligomeric structure alignment, US‐align performs multiple structure alignment (MStA), superimposing three or more structures to obtain an alignment of sequences based on structural superimposition, and can also be used for template‐based docking (Zhang et al. 2022).

GTalign is another major modification of this algorithm reimplemented with GPU support, making it orders of magnitude faster compared to the original TM‐align while maintaining similar accuracy (Margelevicius 2024). Because of the large number of available protein structures and accurate structural models in the post‐AF era, this is a particularly important development allowing for a fast search of large databases, which for DALI or the original TM‐align can take several days. GTalign offers flexibility to find the optimal tradeoff between speed and accuracy, and multiple options for comparing structures, searching databases, and clustering.

SPalign is another sequence‐independent algorithm for protein structure alignment that optimizes the SP‐score, a size‐independent metric analogous to the TM‐score but employing a fixed distance cutoff of 4 Å and an improved normalization by an effective alignment length to better handle varying protein sizes (Yang et al. 2012). A recent modification, SPfast, utilizes a coarse‐grained representation of secondary structure segments, a prefilter with segment‐level alignment and block‐sparse optimization for refinement, and a final atomistic alignment (Liftin et al. 2025). The modified algorithm enables orders of magnitude acceleration, maintaining equivalent sensitivity, suitable for searching large databases. SPfast excels in multi‐domain and partial structure matching, making it highly suitable for high‐throughput searches against AFDB‐like large databases of predicted protein structures.

Despite the high sensitivity of protein structure comparison methods, such as DALI or TM‐align, their utility is limited, especially when applied to large structural databases, because the use of spatial coordinates, or distance matrices, substantially reduces the speed of constructing and comparing protein structure alignments compared to sequence alignments (Kolodny and Linial 2004; Poleksic 2009; van Kempen et al. 2024). Coordinates are more complex objects than linear sequences that can be prefiltered using k‐mers, accelerating the process by orders of magnitude. Moreover, with a local sequence alignment, the search can continue for the remaining part of the sequences without disturbing already aligned regions. In the case of structural alignment of rigid proteins, changes in one part of the protein affect all other coordinates, allowing only global alignment. In most cases, including TM‐align and DALI, this problem is solved by iterative or stochastic optimization.

A linear representation of the 3D protein structure implemented in Foldseek made structure comparison as fast as sequence comparison (van Kempen et al. 2024). Earlier attempts to develop structural alphabets were made using patterns of backbone fragments, usually of 3–5 residues, representing different secondary structure elements, where each C_α_ corresponds to one letter in the sequence. Tools based on this approach, such as 3D‐BLAST, enable faster search but are substantially less accurate compared to traditional structure alignment tools (Mavridis and Ritchie 2010). The structural alphabet implemented in Foldseek (3Di alphabet), in contrast to other structural alphabets that describe backbone conformations, is based on local contacts and their surrounding conformations. For each residue, *i*, its closest spatial neighbor, *j*, and the backbone conformation around these two residues are described, representing the local tertiary structure. A single state describing a residue contains a 10‐dimensional feature descriptor which includes the distance to its closest C_α_ atom and description of its nationhood. Descriptors representing different backbone configurations are discretized into 20 distinct states (tokens), forming the 3Di alphabet. Experiments with larger alphabets containing more tokens did not yield dramatic gains in sensitivity for Foldseek. An alphabet of size 20 was selected as a tradeoff between the amount of encoded information and the accuracy and convenience of *k*‐mer generation, which is crucial for fast prefiltering.

The previous structural alphabets overrepresented some states; for instance, alpha‐helices and beta‐strands were denoted by a single letter that conveyed no information on their neighborhood topology, for example, a beta‐strand direction in a beta‐sheet. The 3Di alphabet encodes more representative information, with higher information density for the conserved hydrophobic core of a protein compared to the more variable surface area. As a result, Foldseek is only slightly less accurate than DALI or TM‐align, but is dramatically, 4–5 orders of magnitude, faster, enabling the search of millions of proteins in minutes and opening new opportunities for systematic comparison of protein structures at a large scale.

The reduction of the possible states of a structural alphabet into a condensed alphabet with 20 letters, as implemented in Foldseek, made it convenient to use structural alphabets in the same way as regular protein sequences. However, condensing the extremely diverse structural states into 20 groups unavoidably leads to an increase in entropy and a reduction in the accuracy of each state that encodes several possible conformations. The authors of Foldseek showed that clustering the structural states into more than 20 groups results in an incremental improvement in performance. However, this could be the result of the implemented algorithm and the relatively small training set (Edgar 2024). An alternative approach was implemented in Reseek, which can use an arbitrarily large alphabet, and therefore, each state in that alphabet can be more informative by encoding more features (Edgar 2024). For each C_α_, the structural alphabet implemented in Reseek encodes several types of information represented as feature vectors (FV). These include information about the amino acid, its C_α_ atom's closest neighbor in space (NEN, Nearest Euclidean Neighbor), and another closest C_α_, but pointing in the opposite direction (REN, Reverse Euclidean Neighbor). The neighborhood of each C_α_, in contrast to 3Di in Foldseek, is described as pairwise distances between all C_α_ atoms of neighboring residues, which captures information about secondary structures, and in the same way, the local conformation is represented by the information on NEN and REN. The FVs are condensed into discrete feature vectors (DFV) of length 16 except for amino acids for which DFV of length 20 are used. A log‐odds score matrix for these discrete features was trained using pairwise alignments from SCOP40 with a TM‐score between 0.6 and 0.8, and log‐odds scores are used in combination with the U‐sorting algorithm (Edgar 2010) for accelerated k‐mer search. Reseek runs at a comparable speed to Foldseek while reportedly outperforming DALI and TM‐align in sensitivity (Edgar 2024).

Foldmason (Gilchrist et al. 2024) and MUSCLE‐3D (Edgar and Tolstoy 2024) are new tools for multiple structure alignment (MStA) based on sequences of structural features that allow phylogenetic analyses of distantly related proteins employing Foldseek and Reseek alphabets, respectively. Although similar methods were available before, Foldmason and MUSCLE‐3D made substantial improvements, making MStA more applicable. First, the flexible alignment, thanks to the structural alphabet that represents protein structure as a sequence, allows accurate superposition of multidomain proteins, even if the homologous domains are displaced in the relative space. Second, both Foldseek and Reseek are several orders of magnitude faster than other similar methods for building MStA, which makes it possible to work with large alignments containing thousands of sequences.

### 3.2 Protein language models and embeddings in homology search

Representation of protein 3D structure as 1D sequence, initially intended for structure comparison, has also found applications in protein language models (pLM). The ESM3 family of generative protein language models uses tokenized protein structures for training, along with protein sequences and functions (Hayes et al. 2025). The structural alphabet implemented in ESM3 is based on 16 nearest neighbors for each C_α_, which are then clustered into one of 4096 structure tokens with a VQ‐VEA autoencoder. Notably, this approach allows encoding the protein structure as a sequence and then decoding it back into structure, with backbone RMSD <1 Å. The ability of generative models to reason about protein structures demonstrates the potential of such models for designing a new generation of homology detection tools. One of the first successful implementations of a bilingual protein language model with a structural alphabet is ProstT5, trained on amino acid and 3Di sequences introduced by Foldseek (Heinzinger et al. 2024). Trained simultaneously in two modalities, ProstT5 generates 3Di sequences directly from amino acid sequences, skipping the intermediate structure prediction step, and hence dramatically accelerating the structure similarity search.

The generation of 3Di sequences from amino acid sequences has multiple advantages, but it depends on Foldseek and requires a target database of structures, or at least 3Di sequences, for search. Amino acid sequence embeddings also can be compared to each other directly, providing a basis for sensitive homology detection without structure prediction (Kilinc et al. 2025). pLM‐BLAST utilizes embeddings from pLM ProtT5 and uses per‐residue similarities to construct global or local alignments, employing a BLAST‐like search approach with specialized substitution and scoring matrices (Kaminski et al. 2023). Comparison of information enriched embeddings provides sensitivity comparable to profile‐profile alignment tools, such as HHsearch, while avoiding the MSA construction steps. Although this approach provides the advantage of local alignment, it also constrains the search speed. It is possible to calculate the similarity of embeddings directly without using similarity matrices and dynamic programming, as implemented in several other tools, thereby accelerating the search by orders of magnitude. TM‐vec is a neural network trained to predict TM‐score from sequences (Hamamsy et al. 2024). This is achieved by per‐residue level sequence embeddings with ProtT5, which are then passed to a twin neural network producing a flattened vector representation. This final representation can be compared using cosine distance to predict TM‐scores. Thus, TM‐vec can predict a TM‐score with a single forward pass, providing an extremely fast route for approximated structure comparison for any pair of sequences. The auxiliary module, DeepBLAST, provides optional structure‐aware sequence alignments that utilize per‐residue embeddings and dynamic programming. A similar approach is implemented in PLMSearch, which employs embeddings from ESM‐1b to directly predict TM‐scores for pairs of sequences (Liu et al. 2024). However, the search is additionally accelerated by a prefilter that sorts out proteins from the target databases that share the same Pfam Clan domains as the query, if these are identifiable in the query sequence; otherwise, the query is searched against the entire database. Finally, the top hits are retrieved for a more robust alignment using per‐residue embeddings in an approach similar to pLM‐BLAST.

Progres (PROtein GRaph Embedding Search) is another method for protein structure comparison that uses embedding instead of explicit coordinates. However, in contrast to the previously described pLM methods, which use sequence embeddings, Progres utilizes a low‐dimensional, sequence‐independent representation of protein structures (Greener and Jamali 2025). In this case, the protein structure, represented as a graph with C_α_ atoms treated as nodes, is embedded independently of its amino acid sequence using a graph neural network (GNN). Each node also contains information about the number of neighboring residues within a 10 Å radius, the torsion angle between four sequential C_α_ atoms, and encoding for residue position in the domain. The final embedding is a 128‐dimensional vector that can be compared to a database of pre‐computed embeddings. The authors experimented with embeddings of different sizes and demonstrated that a 128‐dimensional embedding outperforms state‐of‐the‐art methods in sensitivity for fold searching benchmarks. Despite not providing explicit structural alignment, this approach is extremely fast and accurate, allowing for the comparison of hundreds of structures against large databases such as AFDB and TED in a couple of minutes. The model is trained on single domains and is not expected to work well with multidomain proteins or protein complexes. However, there are multiple methods for splitting proteins into domains based on structural information, and Progres has a built‐in option for the segmentation of multidomain proteins in order to search each domain separately.

### 3.3 What have we learned about the protein universe thanks to the new, AI‐based approaches for structure prediction?

Upon the advent of AF, protein structure comparison almost immediately became a routine method for similarity search, now playing the same role as BLAST plays for sequence comparison. The discoveries of distant homologous proteins with non‐trivial biological implications made by these approaches are far too numerous to discuss here in any detail, so we only mention a few examples, primarily, the largest scale analyses. A recent DALI search in AFDB revealed 100 cases of distant homology; in particular, 35 domains of unknown function (DUFs) were linked to functionally characterized protein families in the PFAM database (Holm et al. 2023). Foldseek was used to cluster more than 214 million structures represented in AFDB in an attempt to assess the true diversity of the available protein structures (Barrio‐Hernandez et al. 2023). This analysis identified 2.3 million clusters and about 13 million structures predicted with low confidence that could not be included in clusters. This structural clustering reduced the number of representative proteins by an order of magnitude compared to sequence‐based clustering with MMseqs2 at a sequence identity of 50%, which yielded about 54 million clusters. Further exploration showed that at least 31% of the clusters were not annotated, but the actual number of uncharacterized proteins in AFDB might be considerably greater given that many annotations, such as the numerous DUFs, provide no biologically relevant information. This study also showed that, although many protein clusters were conserved across all three domains of life, there were also many species‐specific clusters, possibly representing cases of de novo protein birth. In another study, Foldseek, in combination with other tools, was used to classify the domains of all proteins in AFDB, resulting in The Encyclopedia of Domains (TED) (Lau et al. 2024). Although 77% of the non‐redundant domains were found to be similar to one of the domains in the CATH database, this survey also discovered more than 7400 domains with novel folds, considerably extending the boundaries of the chartered fold space. Another large‐scale study leveraged AFDB combined with profile–profile sequence searches to explore the dark matter in the UniProt database, discovering 290 new protein families and at least one novel fold (Durairaj et al. 2023).

Recently, AFDB and ESMAtlas were combined into a single resource, denoted AFESM, that was clustered and explored for different types of novelty (Yeo et al. 2025). Despite the enormous scale of the study, only one new fold was identified, suggesting that the fold space is already (nearly) saturated. In contrast, nearly 12,000 novel domain combinations within the same protein were discovered, primarily, in metagenomic sequences, pointing to a vast, still poorly explored combinatorial space of multidomain protein organizations.

Other work identified specific areas of applicability of AF and, more generally, protein structure similarity search. For example, applying AF2 for structural analysis of proteins encoded by large DNA viruses revealed many cases of recruitment of inactivated enzymes for structural roles, none of which were detectable even with most sensitive sequence search methods (Mutz et al. 2023). Along similar lines, Phold combined ProstT5 and Foldseek to annotate nearly 1.36 million (predicted) phage protein structures in a time comparable to that required for running sequence profile searches on the same scale (Bouras et al. 2025).

Apart from detecting distant, non‐trivial similarities among proteins, a combination of ESMFold and AF allows efficient simulation of the origin of compact protein folds from random amino acid sequences. The first results of such work on in silico evolution show that simple globular folds can evolve with relative ease, requiring a small number of amino acid replacements (less than one per site), compatible with the diversification of protein folds at very early stages of the evolution of life (Sahakyan et al. 2025).

Taken together, these and other studies performed using AF and other AI‐based methods for protein structure analysis leave no doubt about the unprecedented power of these approaches in the analysis of relationships among proteins. However, these methods are still new, and the full extent of the insights into the organization of the protein universe they can provide remains to be investigated.

## 4 DISCUSSION

**FIGURE 3:**
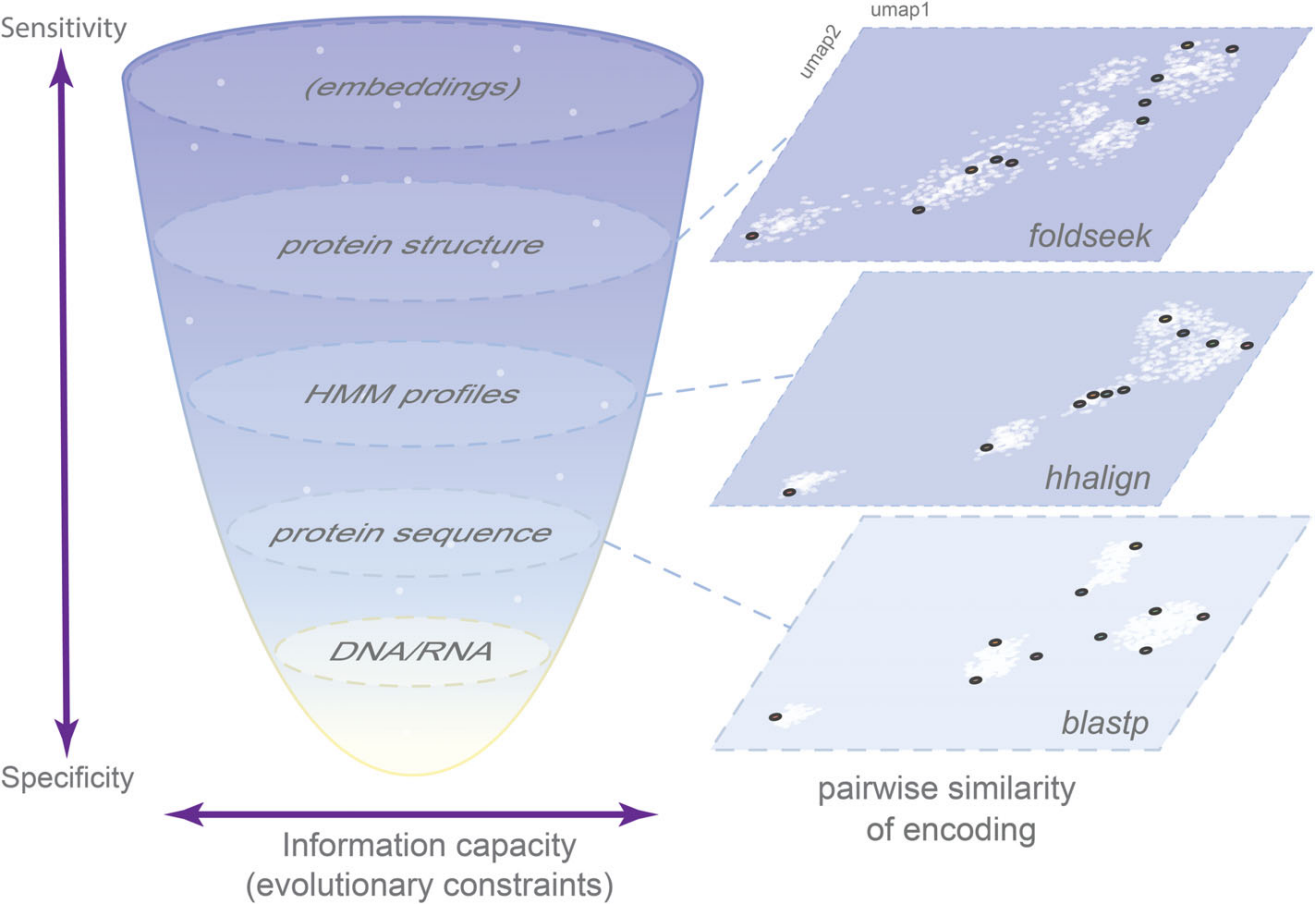
Accuracy of protein comparison methods and their correlation with the information capacity or evolutionary constraints, of protein representation. The funnel on the left shows different representations describing the same protein. Horizontal plots on the right show connections between proteins of the same family (DNA clamp) detected using all‐vs‐all comparison of sequences, HMM‐profiles and structures for the same proteins, and embedding the resulting matrix with UMAP. UMAP projections show pairwise similarities among protein representations of a hypothetical dataset using three methods. Each point corresponds to a hypothetical protein representation (white); black points highlight examples of similar proteins. The increasing clustering from BLASTp to Foldseek conceptually illustrates how higher‐order encodings capture deeper subtler structural similarity and deeper evolutionary relationships that are not apparent at the sequence level. Higher level, particularly, structure‐based, representations are more informative, and methods based on such representations are more sensitive, being able to detect more distant connections, as illustrated by all‐vs‐all companions of the same protein cluster. However, such higher‐level representations have relatively low specificity: For example, it is impossible to detect single nucleotide polymorphisms comparing amino acid sequences and comparing protein folds does not allow detecting amino acid substitutions if those do not change the protein structure.

Proteins can be represented with different levels of abstraction, and as a consequence, protein comparison will have different specificity and sensitivity depending on how detailed the information is in the representation. The selection pressure and evolutionary conservation in proteins follow the direction of the Central Dogma of molecular biology (Crick 1970), from nucleic acids to proteins, whereby amino acid sequences of proteins are more strongly conserved (have less freedom to change) than the protein‐coding nucleotide sequences in which nearly one third of the positions are subject to no or weak selection due to the redundancy of the genetic code (Figure 3). Thus, for revealing homologous relationships among genes, comparing protein sequences (rather than direct comparison of nucleotide sequences) is the approach of choice, a notion that is supported by decades of productive research that culminated in charting the protein universe into numerous families and superfamilies catalogued in databases such as Pfam and InterPro (Paysan‐Lafosse et al. 2025). Each major step in the development of protein sequence comparison, such as the introduction of BLAST to replace FASTA and other earlier methods, PSI‐BLAST and HMM‐based profile comparisons, provided for detection of increasingly subtle similarities among proteins, expanding the observable part of the protein universe. This trend continues with the transition from one‐dimensional protein sequences to three‐dimensional protein structures. As multiple nucleotide sequences can represent the same protein sequence, numerous protein sequences correspond to (nearly) the same structure, which is the final functional form of the information contained in the genes and thus is strongly constrained by selection. Therefore, structural comparison is the ultimate, most sensitive approach for detecting homologs, but possibly, also, similar structures evolving convergently. It has to be kept in mind, however, that comparisons at different levels of the information hierarchy have different areas of applicability: protein sequence comparison, for example, cannot detect single‐nucleotide polymorphisms, whereas structure comparison is not useful for analyzing specific amino acid replacements.

Protein structure can be represented in a variety of ways, such as spatial coordinates and distance matrices, but also structural alphabets that are discrete and more general than the former, providing for much faster protein comparison. Another promising way of representing proteins is embeddings created with protein language models (pLM), which can store diverse information about protein sequences and structures. Successful attempts have already been made to use pLM embeddings for protein comparison (Hong et al. 2024; Iovino et al. 2024; Kaminski et al. 2023; Liu et al. 2024).

Although structure comparison is the most robust and sensitive approach for detecting similarity between proteins (Holm 2020; Holm 2022; Holm et al. 2023; Illergard et al. 2009), it is insufficient to decipher phylogenetic relationships, especially among proteins that share high structural similarity. However, a structural alignment helps create an accurate sequence alignment which otherwise might not be feasible to obtain for distant homologs. Such alignments can then be used further for phylogenetic analysis operating on the developed evolutionary models. However, much caution is due when applying current phylogenetic methods to a structure‐guided amino acid alignment if the analyzed proteins diverged to substitution saturation. A promising alternative direction is to develop substitution matrices for a 3D structural alphabet which would allow phylogenetic inference to be made directly from structural comparisons holding potential for deciphering the evolutionary history of highly diverged protein families that are currently not amenable to phylogenetic analysis. Several attempts to develop substitution matrices for the structural alphabet implemented in Foldseek and to apply these to construct phylogenies have already been made (Garg and Hochberg 2024; Moi et al. 2023; Puente‐Lelievre et al. 2024). Structural phylogenetics is still in its infancy but can be expected to become an indispensable approach for deciphering deep evolutionary relationships.

Homology inferences from structure alignments should be treated with caution in cases when proteins share simple folds with few structural elements or with repeating patterns. In particular, simple beta‐sandwiches and helix‐turn‐helix folds sometimes share significant structural similarity but no detectable sequence similarity, even when estimated from a structural alignment. This can be the consequence of divergent evolution reducing the sequence identity to an undetectably low level, whereas the fold changes relatively little. However, such structural similarities also could result from convergence, that is, independent evolution of similar (analogous as opposed to homologous) structures from unrelated sequences (Orengo et al. 2001). Convergent evolution is especially pertinent for small proteins with simple folds, when two proteins adopt the same structural configuration due to its thermodynamic stability and/or evolutionary accessibility whereby that fold serves as an attractor in the sequence space (Johnston et al. 2022; Wolynes 1996). Structure comparison assigns a high similarity for such non‐homologous but similar folds, posing the difficult problem of distinguishing homology from convergence for which there is currently no general solution (Johnston et al. 2022; Murata et al. 2024; Orengo et al. 2001; Wolynes 1996; Wright 2025).

The search for homologs in practice is a score cutoff selection problem as a trade‐off between sensitivity and error rates. The gold standard solution for these problems was the E‐value introduced by BLAST, later adopted by profile HMM methods, and now by structure search methods. For sequence comparisons, the E‐value is derived from the Gumbel extreme value distribution, which models the alignment scores of random sequences. Recently, this problem was revisited with a focus on the significance estimation of structure search algorithms, and it was shown that for structural alignments, the Gumbel distribution does not accurately fit the high‐scoring tail, leading to significance overestimation (Edgar and Sahakyan 2025). This observation reinforces the notion that algorithmic and methodological improvements for homology search must be accompanied by appropriate statistical procedures for significance estimations, the approach that made BLAST revolutionary in the early era of sequence searches.

The rapid advance of pLM and their application in remote homology detection is transformative in many aspects and opens new avenues for further development of even more powerful tools. Nevertheless, in practice, pLMs are reliable for large, well‐studied protein families, but frequently fail for singletons and uncharacterized groups of proteins that are particularly abundant among viral and metagenomic sequences. In this context, traditional sequence‐based approaches may be advantageous because they do not rely on transformers or other generative models and are not biased with respect to the nature of the query sequences (Zhang et al. 2025). It remains to be seen whether more generic but nevertheless efficient pLMs can be developed in the future.

## 5 CONCLUDING REMARKS

The recent decisive progress in fast and accurate protein structure prediction complemented by the increasingly efficient methods for structure comparison has radically transformed the study of the protein universe. Comprehensive comparative analysis of the structures of all known proteins has become a realistic task although structural comparison remains computationally intensive and insufficiently precise. Development of a new generation of better theoretically grounded, robust and efficient protein structure comparison methods that would do for structure analysis what BLAST, and then, HMM‐based tools did for sequence analysis remains a key goal. A related crucial goal is the development of evolutionary models for protein structures which will pave the way to robust and efficient methods for structural phylogenetics. Within a few years, such approaches can be expected to provide for charting the protein universe at the level of structures which is the most adequate representation of proteins.

## AUTHOR CONTRIBUTIONS

**Harutyun Sahakyan:** Conceptualization; writing – original draft; writing – review and editing. **Pascal Mutz:** Writing – original draft; writing – review and editing. **Victor Tobiasson:** Writing – original draft; writing – review and editing. **Eugene V. Koonin:** Conceptualization; writing – original draft; writing – review and editing.

## CONFLICT OF INTEREST STATEMENT

The authors declare no conflicts of interest.

## Data Availability

Data sharing not applicable to this article as no datasets were generated or analysed during the current study.
